# Combining Optimal Control Theory and Molecular Dynamics for Protein Folding

**DOI:** 10.1371/journal.pone.0029628

**Published:** 2012-01-06

**Authors:** Yaman Arkun, Mert Gur

**Affiliations:** 1 Department of Chemical and Biological Engineering, Koc University, Istanbul, Turkey; 2 Center for Computational Biology and Bioinformatics, Koc University, Istanbul, Turkey; National Institute for Medical Research, Medical Research Council, London, United Kingdom

## Abstract

A new method to develop low-energy folding routes for proteins is presented. The novel aspect of the proposed approach is the synergistic use of optimal control theory with Molecular Dynamics (MD). In the first step of the method, optimal control theory is employed to compute the force field and the optimal folding trajectory for the 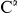 atoms of a Coarse-Grained (CG) protein model. The solution of this CG optimization provides an harmonic approximation of the true potential energy surface around the native state. In the next step CG optimization guides the MD simulation by specifying the optimal target positions for the 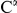 atoms. In turn, MD simulation provides an all-atom conformation whose 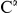 positions match closely the reference target positions determined by CG optimization. This is accomplished by Targeted Molecular Dynamics (TMD) which uses a bias potential or harmonic restraint in addition to the usual MD potential. Folding is a dynamical process and as such residues make different contacts during the course of folding. Therefore CG optimization has to be reinitialized and repeated over time to accomodate these important changes. At each sampled folding time, the active contacts among the residues are recalculated based on the all-atom conformation obtained from MD. Using the new set of contacts, the CG potential is updated and the CG optimal trajectory for the 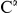 atoms is recomputed. This is followed by MD. Implementation of this repetitive CG optimization - MD simulation cycle generates the folding trajectory. Simulations on a model protein Villin demonstrate the utility of the method. Since the method is founded on the general tools of optimal control theory and MD without any restrictions, it is widely applicable to other systems. It can be easily implemented with available MD software packages.

## Introduction

After their synthesis in the cell, proteins fold to their unique native states in order to fulfill their biological functions. Significant amount of research has been devoted to the determination of the alternative folding routes that bridge the denatured and native protein configurations. Recent studies show that, the folding landscape is rugged and funnel-shaped, and the protein prefers to follow the folding routes that minimize its energy [1], [2]. At the same time proteins avoid those pathways that result in high-entropy loss [3], [4].

In general coarse-grained mesoscopic models are used to facilitate the protein folding process. At the same time these simplified models provide useful physical insight before embarking on full scale modeling. At the heart of coarse-graining lies the “lumping” of atoms to fewer interaction sites (e.g. 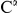 atoms in the case of proteins). When coarse-grained (CG) models are combined with more refined atomistic models, important headway into the problem of protein folding can be made [5]. For example [6] used CG models to identify physically meaningful starting conformations (instead of extended initial structures) for the MD simulations of the protein folding process.

Recent multiscale or multigraining methods combine CG models with higher resolution models in molecular simulations [7]–[10]. For the folding problem it is important to note that CG models must be constructed to preserve the dominant characteristics of folding without significant loss of accuracy. To this end the potentials of mean force for CG models have been designed by matching the radial distributions of CG and atomistic models using the iterative Boltzman technique [11], [12]. In [13] a force matching method has been presented to construct a CG model that has a mean force field which matches the ab initio MD reference forces.

There is ample evidence in the literature that folding dynamics are governed by a reduced dimensional manifold that consists of slow/low-frequency modes. These modes persist over long time scales and influence the conformational changes and the protein's function, while the rest of the modes reflect the localized high-frequency dynamics [14], [15]. Significant reduction in dimensionality is basically due to the interresidue correlations which result from the contacts made during folding. In strong support of this observation, it has been shown that the motion of the backbone 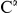 atoms explains most of the essential folding dynamics [16], [17]. This further justifies the use of reduced order CG representations for the characterization of folding dynamics.

Folding can be characterized as a dynamical process during which the protein starts from a random unfolded configuration and folds into its unique native state under the action of inter and intramolecular forces. This physical process has lent itself into different types of mathematical formalisms in the past. One approach is called the action-derived molecular dynamics (ADMD) which solves a two-point boundary value problem [18]. In this work the authors discretize the action (Lagrangian) over time along possible trajectories that satisfy Newton's equation of motion subject to preassigned initial and final conditions. Minimization of this action generates the folding pathway. Optimal control is yet another natural approach to formulate and solve the folding problem. Our earlier work [4], [19] has contributed in this direction by applying control theory to linear CG representations of proteins. However direct application of optimal control to the nonlinear atomistic models used in MD is computationally prohibitive as time scale and the number of residues increase for realistic problems.

As an alternative, in this paper, we have combined the best of two worlds of CG modeling and MD. Performing dynamic optimization using a CG model provides simplicity and speed whereas MD supplements accuracy. This is the motivation behind the proposed method. Specifically we are interested in developing a method that can easily compute low-energy folding trajectories and at the same time closely represent the real protein. To this end we utilize the well-founded machinery of optimal control theory to compute the folding trajectory for the 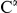 atoms. This is coupled with MD which performs all-atom dynamic simulation and refines the CG optimal folding trajectory. This CG dynamic optimization and MD refinement cycle is repeated at sampled folding steps until the protein reaches its native state. We now describe each element of the method in detail below.

## Methods

### Coarse-Grained Model

In the CG model each residue is represented with a spherical bead centered at the 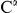 atom. The position of the i-th bead in space is denoted by the vector 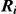 with respect to a fixed reference frame. Beads are connected with each other with springs. Beads-and-springs representation of the protein is common in the literature [20]. The total position vector is defined by 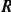, whose i-th entry is the position vector for the i-th bead 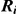. Folding dynamics is governed by the equation of motion:
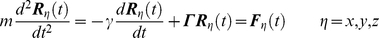
(1)where, the subscript 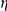 denotes the *x*, *y*, or *z* coordinates; *m* is the mass of the i-th residue; 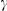 is the local friction constant; 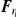 is the force field; and 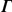 is the connectivity matrix that represents the covalent bonds of the initial protein structure. Assuming that the mass term is negligible [20], [21] and expressing the variables in deviation from their native state values leads to
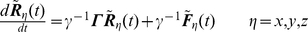
(2)where 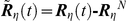, and 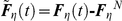, and the superscript *N* denotes the native state. In the following, in the interest of simplicity, we omit using the subscript 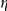 that refers to the *x*, *y* or *z* coordinates. We now formulate the dynamic optimization problem as an optimal control problem.

### Optimal Control Formulation: Linear Quadratic Regulator

The CG dynamic model that governs the motion of the backbone is given by:
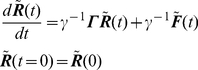
(3)It follows from optimal control theory [22] that the Linear Quadratic Regulator (LQR) synthesizes a feedback solution for the force field 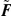 that drives the initial state 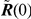 to the desired zero-state. This means that the unfolded initial structure folds to its native state under the optimal force field designed by LQR. Among many possible trajectories that satisfy Eq. 3, LQR chooses the one that is optimal with respect to a prescribed objective function. Specifically the following optimization is solved subject to Eq. 3:
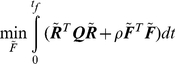
(4)Since the protein tends to move downhill on the energy landscape, the first term under the integral represents the potential to be minimized as it is shown below.

The contact map of a protein is an *n*x*n* matrix defined by:
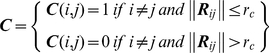
(5)where 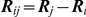 denotes the pair distance vector from residue *i* to residue *j*.

The parameter 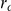 is the cut-off distance (e.g. 7 Å) for a contact to be established between two residues. The Laplacian matrix [23] is an *n*x*n* matrix constructed from the contact map ***C*** as follows:
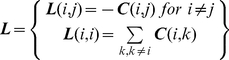
(6)When 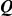 is equated to the above Laplacian matrix excluding the covalent bonds (i.e. 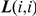), one gets [4]:
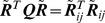
(7)which is in the form of an “harmonic pair potential” centered at the native state [24]. Minimization of this potential over the folding time horizon 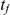 generates the optimal force field that folds the backbone of the protein. At the same time, energy cannot decay to zero infinitely fast by using an unrealistic, unbounded force field which would violate the principle of minimum entropy loss. Thus, a second term is included in the objective function (4) to avoid such trajectories. The parameter 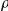 associated with this term acts like a Lagrange multiplier to penalize entropy losses. Typically it is used as a tuning parameter to reflect the relative significance of the two terms under the integral.

It is well known that the folding mechanism is encoded in the topology of the native state and the Hamiltonian function of the protein [6], [25]. For these reasons numerous unfrustrated models have been built based on the topology of the native state. As a zeroth order approximation, these models ignore the nonnative interactions [26]. Recognizing that the nonnative interactions can play a role in the earlier stages of folding, [27] has introduced a minimalist model which includes the nonnative interactions through a nonlocal potential. In our method we compute the contacts made at each folding step and update the contact matrix 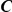, Laplacian 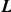 and 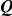 accordingly. Thus nonnative contacts are incorporated into the CG model and optimization, if they happen to form temporarily during folding.

In Eq. 3 the connectivity matrix 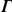 has all negative eigenvalues but one zero eigenvalue. This zero eigenvalue needs to be stabilized by the optimal controller so that the protein asymptotically can reach its native state. To do so 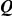 must be positive definite. However when 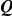 is set equal to the Laplacian, it becomes nonpositive definite since the Laplacian matrix has all positive eigenvalues but one zero eigenvalue by definition. Therefore 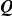 is modified accordingly:
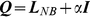
(8)where 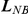 is the Laplacian excluding the covalent bonds; the parameter 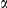 is a small positive number, and 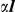 is added to make 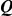 positive definite and guarantee stability.

As the terminal time 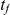 approaches infinity, the optimal solution to the above optimization problem is given by a negative constant feedback control law:
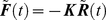
(9)where 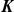 is a constant matrix that is easily computed using the algebraic Riccatti equation [22].

Note that when a random force 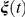 in the form of white noise is added to the right hand side of the CG model i.e. Eq.3, the equation of motion follows the Langevin dynamics. In this stochastic case, the feedback law given by Eq. 9 is still optimal as it now minimizes the expected value of the objective function.

### Synthesis of the Optimal Harmonic Potential for the CG Model

The above structure of 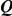 imposes a similar structure on the optimal feedback gain matrix 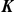. In other words, the optimal 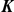 can be decomposed similarly to Eq. 8:
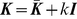
(10)

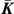
 is the “harmonic spring constant matrix” with its row sums equal to zero. The entries of 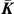 represent the springs connected between the residues and their values are the corresponding spring constants. These values are not a priori selected but are optimally assigned by the optimal controller. The second term 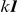 represents the springs that connect the residues directly to their native states. For these connections, spring constant values are all the same and equal to 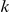. These additional connections are necessary to stabilize the translational motion due to the zero eigenvalue.

**Figure 1:**
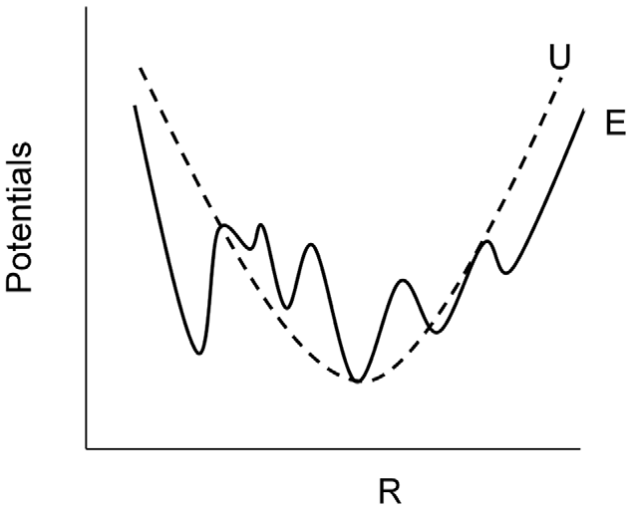
One dimensional schematic of potential energy surfaces. *U* is the harmonic CG potential; *E* is the protein's true potential. Native values are subtracted from both.

Since the optimal force field 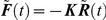 and 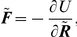 the optimal controller has effectively synthesized the following optimal harmonic potential:
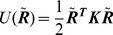
(11)This potential is a CG approximation of the true potential energy surface around the native state as shown in Fig. 1. It is parametrized through 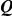 since 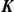 depends on 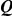.

When the optimal force field 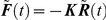 is implemented, the CG dynamical model i.e. Eq. 3. becomes:
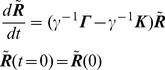
(12)It is this dynamical manifold that governs the motion of the alpha carbons.

### Interfacing the CG Model Based Optimization with MD

**Figure 2:**
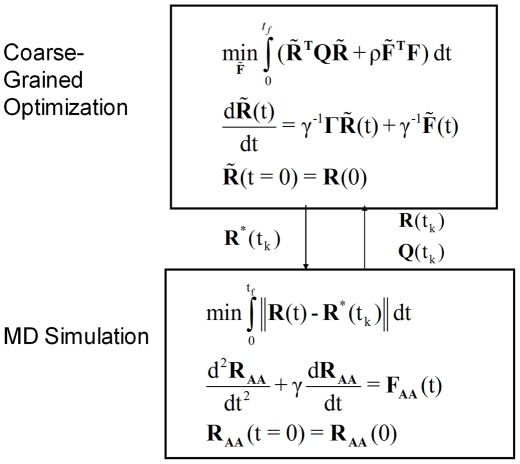
Block diagram representation of the method.

The novel aspect of the proposed method and the main contribution of this paper is the concerted use of CG dynamic optimization and MD. CG optimal folding trajectory guides the MD simulations. In return the results from MD are used to refine the CG trajectory by making the necessary adjustments. The block diagram representation of the method with the information exchange between CG optimization and MD tasks is shown in Fig. 2. Implementation of the method is explained next.

**Figure 3:**
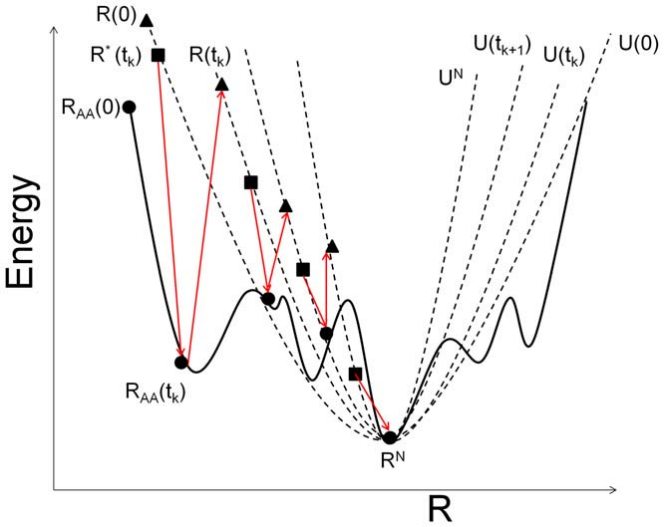
Exchange between the updated CG potentials and the true all-atom MD potential. Dashed parabolas are the CG potentials that are updated. Arrows show the hopping between potentials at different sampling times 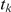, 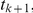 etc. until the native state is reached. Triangle represents the initial state in each CG optimization. Square represents the optimal target 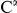 conformation. Circle represents the all-atom structure reached after MD refinement. Triangle-square-circle sequence is one computational cycle of CG optimization and MD refinement.

For an initial unfolded structure the position vector for all the atoms i.e. 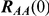 is available (see Fig. 3). The position vector for 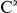 atoms i.e. 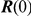 is extracted from this 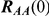 . The Laplacian for the initial structure is computed from its contacts and 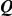 is initialized as in Eq.8. Next LQR computes the first optimal CG trajectory for the 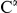 atoms. Denote this trajectory by 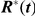, where “*” indicates that it is optimal for the CG model.

Now pick a particular time 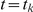, and sample a conformation 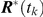 from the first optimal CG trajectory 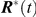. This conformation is the first “target” structure for MD. It is supplied to MD as shown by the first arrow going down in Fig. 3. Next targeted molecular dynamic (TMD) simulation, which has the 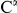 positions of the optimal target structure 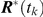 as its target, is performed. In order to relieve any stress/strains that may have occured by forcing the 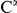 positions to the CG predicted positions via TMD, a succesive short equilibration (Conventional MD) to the TMD simulation is performed. It has to be noted that continuity in the all atom simulations is achieved by starting the TMD simulations from the final structures of the previous equilibration simulations.

In essence the following type of minimization is solved (see MD block in Fig. 2):
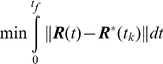
(13)This is implemented by performing targeted molecular dynamics (TMD). TMD has been used in the past to accomplish large conformational changes by using a bias potential or harmonic restraint in addition to the usual MD force field [28], [29]. We have implemented TMD within NAMD software package [30]. At each time step, NAMD computes the force on each atom from the gradient of the bias potential given by
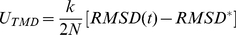
(14)where 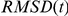 is the root mean square deviation of the current conformation from the native structure and similarly 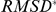 is the the root mean square deviation of the target conformation from the native structure. 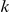 is the spring constant and 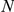 is the number of targeted atoms.

TMD is followed by equilibration of potential and kinetic energies. The resulting structure 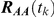 is an all-atom stable structure whose 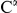 positions are closest to the CG optimal structure that was targeted.

After MD simulation, two pieces of information are supplied to CG optimization. This feedback information includes the new 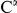 position vector 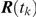 and the new matrix 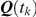. This is shown by the arrow up (feedback) from MD to CG optimization blocks in Fig. 2. By definition the entries of 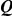 are determined by 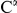 atoms that make contact. Since these contacts change during the course of folding, 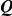 must be updated after each MD. Next, time is advanced to 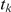, and CG optimization is repeated with the new initial state vector 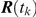 and the new 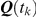. This cycle of CG dynamic optimization and MD feedback correction is repeated until the end of folding. At the end one obtains an optimal folding trajectory that consists of *N* conformations with full atomic details:
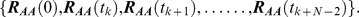



CD optimization-MD cycle generates the folding trajectory by “hopping” between the approximate CG harmonic potential i.e. Eq. 11 and the true potential surface. During this hopping, the CG harmonic potential guides MD by providing the target conformations to explore in full detail. At the same time the local information from MD updates the CG potential. When this learning cycle is repeated over time, convergence to the native state is accomplished. The “hopping” between the potential surfaces is schematically illustrated in Fig. 3. Dashed parabolas represent the CG potentials 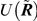. These potentials get updated after each MD simulation. As more contacts are established, the potentials get narrower as shown. This enhances the convergence to the native state. The solid curve represents the true MD potential. Arrows show the hopping between potentials that occurs at different sampling times 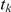, 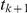, etc. until the native state is reached. The CG minimizer-the Linear Quadratic Regulater itself has a global minimum since the model is linear and the objective function is quadratic. But the folding energy landscape has many local minima and global search over this multi-dimensional surface is problematic. For this reason this surface is approximated by the CG optimization and this approximation is repeated along the folding trajectory. In this sense the trajectory is a collection of local optimums.

The chicken Villin headpiece, Protein Data bank code 1VII.pdb, was selected as an example to demonstrate the proposed method. Villin has 36 residues and it is one of the fastest folding and stable proteins. Due to its small size and short folding time, Villin has been the subject of several theoretical and experimental investigations [31]–[36]. Unfolded starting structures were constructed in Hyperchem by first generating the whole structure as a beta sheet and then geometrically optimizing the structure using the the Polack-Ribiere Conjugate Gradients algorithm. Folded native structures on the other hand were selected from the pdb bank. All molecular dynamics (MD) simulations were performed for an NVT ensemble in explicit solvent (water) using NAMD 2.7b package with CHARMM27 force field at 310 K. Villin, both in its folded and unfolded form, was aligned with the x-axis using the transformation matrix required to bring the vector between the first 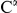 atom and the last 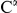 atom to the x-axis. Folded Vilin was solvated in a waterbox of 45 Å cushion in the x- direction, 15 Å cushion in the y- direction and 15 Å cushion in the z- direction. Unfolded Villin was solvated in a waterbox of 7 Å cushion in the x-direction, 15 Å cushion in the y- direction and 15 Å cushion in the z-direction. Ions were added in order to represent a more typical biological environment. Periodic boundary conditions were applied and Langevin dynamics was used. All atoms were coupled to the heat bath. A time step of 1 fs was used. No rigid bonds were used in order to keep all degrees of freedom.

Two minimization-equilibration cycles were applied to the unfolded and folded structures. The purpose of the first minimization is to relax the water and it is performed under NPT conditions. For that purpose the protein was kept fixed throughout the 20000 steps of minimization and 0.5 ns of equilibration. The second cycle was applied under NVT conditions to find a local minimum of the whole system's energy. Again 20000 steps of minimization were performed which was followed by equilibration. In this second cycle the 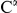 positions of the unfolded structure was fixed throughout the whole simulation so that the structures stayed unfolded. For the folded structures on the other hand the protein was set free to move.

The 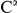 coordinates of the equilibrated unfolded structure (which are essentially the same with the non-equilibrated one) were selected as the starting structure and the 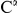 coordinates of the NMR structure, 1VII.pdb, were selected as the final structure to compute the first optimal CG folding trajectory. Dynamic optimization was solved and the resulting CG optimal folding trajectory for 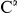 positions was recorded and divided into 50 time steps. The first of these 50 recorded structures was selected as the first target conformation for MD. A 0.01 ns long TMD simulation, starting from the equilibrated unfolded structure, was performed to bring the actual 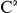 positions of the all-atom conformation to the targeted values. An elastic spring constant of 2000 
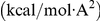
 was used in TMD. After TMD simulation, a 0.05 ns long conventional molecular dynamic (CMD) simulation was performed for equilibriation. The final structure was recorded as the first all-atom MD structure along the folding trajectory. Using this recorded structure as the starting structure of the CG model, a new CG optimal folding trajectory was obtained; a new target structure was chosen and TMD simulation followed by consecutive MD equilibration was performed. These optimization-TMD simulation-MD equilibration cycles, named folding steps, were repeated until enough convergence to the native state was achieved. The final structure to generate all of the CG optimal folding trajectories was selected to be the NMR structure of villin as it was the case for the first dynamic optimization.

## Results

Table 1 summarizes the evolution of the selected target structures as time progresses. Each step in the table corresponds to one of the CG predictions and the successive MD relaxation cycle. In CG optimization we obtain a prediction of the CG folding trajectory. Our aim is to find the closest all atomistic conformations to these predictions. In order not to loose accuracy, steps taken by the CG model should be chosen as small as possible whereas they must be large enough so that the system does not return to its previous state. Initial structures exhibit significant differences as they fold while this difference diminishes as the native state is approached. In other words the RMSD between the starting structure and final structure of the CG folding trajectory decreases with each folding step. Therefore, during the early stages of folding, one should choose the target structures from the beginning structures predicted by the CG optimal trajectories. However, in the later stages, the target structures should be chosen from the structures near the end of the CG optimal trajectories so that large enough RMSD from the starting structure can be obtained to derive the TMD. This is confirmed by the structures noted in Table 1. For the first 4 time steps the first predicted CG optimal structure is used to evolve the conformation in MD. However for advanced steps a further predicted structure is taken which is, for example, the 50^th^ predicted structure for all folding steps after 51.

**Table 1:** Evolution of targeted CG structures.

Step	Target structure selected from the CG folding trajectory
1–4	1
5–9	2
10–14	3
15–19	5
20–24	7
25–29	11
30–34	15
35–39	23
40–44	31
45–50	47
51---	50

**Figure 4:**
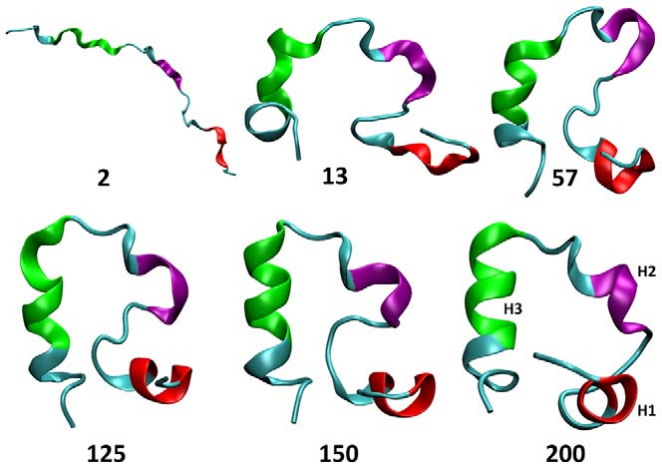
Some sampled conformations on the folding trajectory. Numbers indicate the folding step. H1 (red), H2 (purple) and H3 (green) denote the three helices of Villin headpiece.

Villin has 3 short helices, H1, H2 and H3 surrounding a closely packed hydrophobic core. These helices contain the residues 4–8, 15–18 and 23–30, respectively. They are held together by a loop and a turn. Fig. 4 shows sampled conformations from the folding trajectory obtained by our method. At the early stages of folding, helix H3 is the first one that begins to form which is followed by H1. After an hydrophobic collapse, helices H1, H3 continue to form concurrently. Helix H2 is the last and most difficult one to form which is consistent with the observations made in [34].

**Figure 5:**
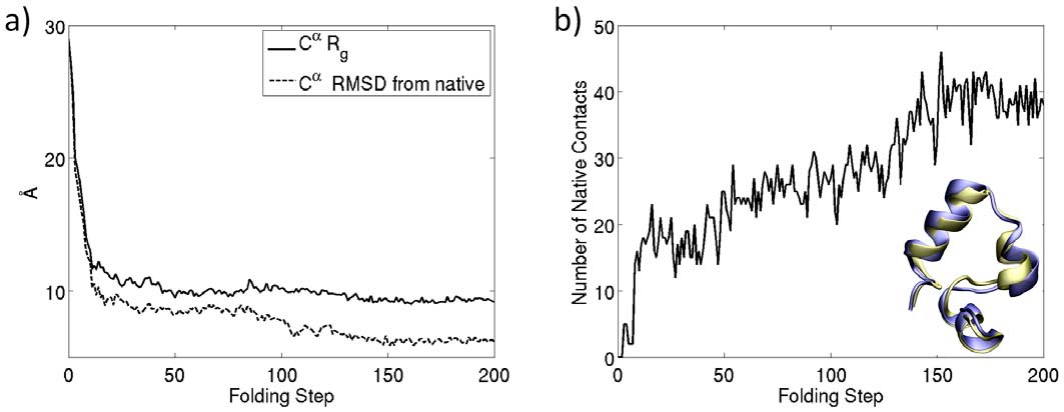
Evolution of radius of gyration, RMSD and contacts during folding. Evolution of radius of gyration 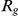, and RMSD of 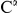 during folding are shown in panel (a). Evolution of number of contacts and comparison of the final MD structure with the native structure appear in panel (b).

At the end of folding, the final MD structure comes very close to the NMR native structure as shown in Fig. 5(b). The RMSD from the NMR native structure for the 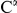 atoms monotonically decreases towards the native state as shown in Fig. 5 (a). After the folding step 150, the RMSD values fluctuate around 1.49 Å, exhibiting a minimum of 1.16 Å. Among different techniques and folding simulations studied in the literature, most of the reported 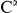
- RMSD values for Villin are >3.0 Å [33]. As an example [35] presents a 200-ns fast folding simulation using the implicit solvent method and reports an RMSD value greater than 3.46 Å. However, more recently, lower RMSD values were obtained. The authors in [33] used the replica exchange MD method and showed that Villin folded consistently to the native state. The lowest 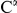
- RMSD from the X-ray structure was given as 0.46 Å [33]. In [34] the action-derived molecular dynamics method (ADMD) was applied and the 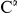
- RMSD from the X-ray crystal structure fell below 1.0 Å. 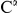
- RMSD values given in Fig. 5 (a) are close to these improved values reported in the literature. Also our RMSD values with respect to the backbone atoms are similar to the 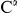
- RMSD values which indicates that the backbone motion of the protein follows the CG optimal trajectory of the 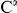 positions further justifying the use of CG optimization.

The initial rapid decrease in radius of gyration 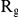 (see Fig. 5(a)) is indicative of the initial hydrophobic collapse and compaction of protein. The slower decay of RMSD in later stages of folding is due to the completion of local secondary structures and the tertiary contacts. The contacts which exist between two residues which are seperated more than two residues in sequence and have a 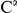-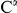 distance smaller than 6.5 Å are shown in Fig. 5(b). Similar to the behavior of RMSD, the number of contacts converges after folding step 150. These trends and numbers are similar to those given in [34].

**Figure 6:**
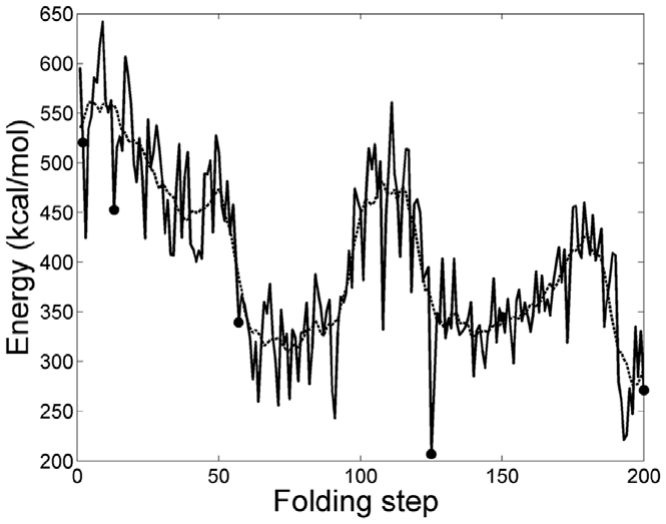
The internal energy of protein during its folding. The dashed curve is obtained by taking moving average of the data and it is included to mark the general trend. Circles correspond to the structures shown in Fig. 4.

**Figure 7:**
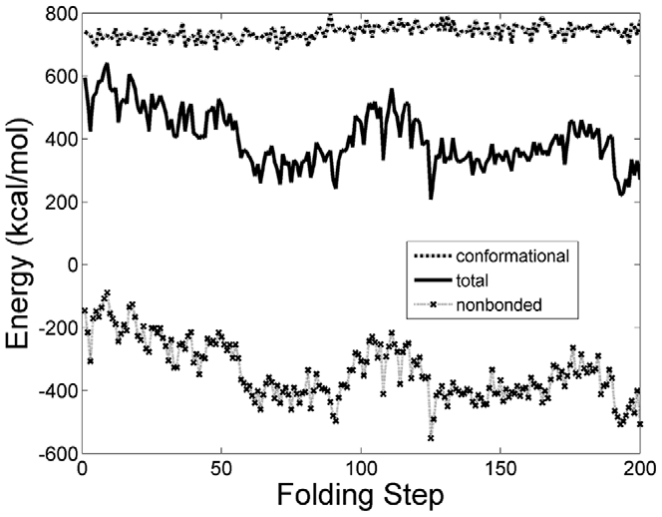
Components of the internal energy. Conformational energy (bonds, angles, dihedrals, and impropers), nonbonded energy (vdW and electrostatic energy), and total energy are compared. Nonbonded energy determines the general trend of the total energy.

All components of the internal energy of the protein (i.e.bonds, angles, dihedrals, impropers, Van der Waals, and electrostatic) were evaluated using the NAMD Energy Plugin in VMD. In Fig. 6 the internal energy profile of Villin for the computed trajectory that consists of 200 folding steps is shown. The sampled conformations seen in Fig. 4 are marked on Fig. 6 by circles. Different energy components are compared in Fig. 7. It is the nonbonded energy (Van der Waals and electrostatic in particular) that determines the general trend of the total energy profile.

Conformational changes during folding have a direct effect on the internal energy. Therefore Fig. 6 shows the protein's internal energy only. The plot does not include solvent-protein ineractions and solvent energies. The internal energy profile exhibits many short time-scale local fluctuations which persist throughout folding. These fluctuations reside on a slower time-scale trajectory (shown by dashed curve) which follows a downward trend towards the native state. In order to explain the energy fluctuations in Fig. 6, we have compared their magnitudes with the magnitude of the equilibrium fluctuations of the native state. For this purpose, we performed a 2.62 ns equilibration of the native structure of Villin headpiece using NAMD, and from the last 0.5 ns of data we computed the root-mean-square fluctuation of energy:
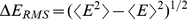
(15)This gave a value of 34.1 kcal/mol. Next we computed 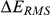 for the local energy fluctuations directly from the simulated folding trajectory of Fig. 6. The results are listed in Table 2. It is seen that the internal energy fluctuations 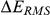 along the folding trajectory are about the same order of magnitude as the value calculated from MD equilibration of the native state (i.e. 34.1 kcal/mol). In the early stages of folding, fluctuations exceed the native state's equilibrium value (see steps 1–124 in Table 2) and as the protein folds to its native state, fluctuations approach the native state's equilibrium fluctuation of 34.1 kcal/mol as shown in the later folding steps (126–200). It can be concluded that fluctuations in Fig. 6 are, almost half of the time, around the same value as the equilibrium value. In addition it is important to note that internal energy fluctuations larger than the equilibrium value can occur along the folding trajectory because folding is an out of equilibrium process during which there is not enough time for all the conformational rearrangements to complete at each folding step. All these factors contribute to the magnitudes and general trend of the MD energy fluctuations along the folding trajectory.

**Table 2:** The internal energy fluctuations along the folding trajectory.

Folding Step Interval in Fig. 5.	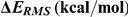
0–56	61
57–100	48
101–124	56
126–170	28
171–185	36
195–200	32

When moving average is applied over time to the internal energy data, the local dynamic fluctuations can be filtered and a slower time-scale folding trajectory is revealed as shown by the dashed curve in Fig. 6. In the early stages of folding we see that the internal energy decays sharply on the average. Here the energy decrease is associated with the significant conformational changes of the backbone (see early structures in Fig. 4.) and this dominates the local events and their energy fluctuations. This result is also in agreement with the initial decay of RMSD and 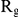 plots in Fig. 5 (a). During the later stages of folding (after step 90), conformational changes become more incremental as the native state is approached while the local fluctuations persist as expected. Finally the energies of our attained conformations at the end of folding were found to be within the energy fluctuations of the native state. For example the energy value of the structure at the 192th folding step is equal to 221.2 kcal/mol. We have performed MD equilibriation on the NMR native structure and found that 221.2 kcal/mol falls within the internal energy fluctuations of the equilibriated NMR native structure.

Above results illustrate the workings of the proposed method and show that the method has successfully produced a folding trajectory that has reached a reasonable neighborhood of the native state for a particular protein. Additional MD simulations can now be afforded to fine-tune, allow for further local rearrangments, and improve the folding, if deemed necessary.

## Discussion

It is now well-recognized that the long term folding dynamics of proteins is governed by a reduced order manifold that is built from the correlated motion of its residues. For this reason low dimensional, simplified CG models have proven to be useful to advance our understanding of folding dynamics while demanding modest computational power. On the rugged and funnel-shaped energy landscape, there exist many alternative folding routes that bridge the denatured and native protein configurations. Among these folding routes, the protein prefers to follow the folding routes that minimize its energy and its entropy-loss. However generation of these folding routes from first principles and computation of the optimal folding routes is not a trivial task. Direct search for the optimal pathway by a dynamic optimization based on a detailed atomistic model is computationally prohibitive for most typical problems at present. Therefore, in this paper, we have introduced a method that makes use of a CG optimization which guides the MDs in search of the optimum folding routes. CG optimization minimizes an harmonic approximation of protein's true potential and constructs the optimal trajectory for the positions of 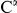 atoms. Subsequently MD refines this optimal folding trajectory at the atomistic level. To this end, we have performed TMD to follow closely the optimal pathway generated by the CG optimization. The positions of 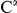 atoms from the CG optimal trajectory are targeted and TMD is able to make the necessary conformational changes. This CG dynamic optimization and all-atom MD refinement cycle is repeated at each sampled folding time until the protein reaches its native state. In doing so the folding route is continuously reoptimized and updated by incorporating the local information obtained from MD. In particular, at each sampled folding time, the contact map of the protein and its harmonic CG potential are updated, and CG optimization is repeated with this new data. The method is computationally attractive and easy to interface with the available MD simulation packages. The method is based on a general conceptual framework which permits the use of different types of CG models and potentials. Different ways to update the CG grain model during folding is open to further research.

We show in our simulation example that the Villin headpiece can be successfuly folded to its native state by the method. Results are consistent with those in the literature. For the proof of concept, we delibaretly chose a small “benchmark” protein such as Villin since it is the most widely studied system in the literature where folding trajectories are available. This allowed us to make comparisons and validate our results. Otherwise the method is widely applicable to larger proteins.
